# Response to Commentary: Long-term Changes of Inflammatory Biomarkers in Individuals on Suppressive Three-Drug or Two-Drug Antiretroviral Regimens

**DOI:** 10.3389/fimmu.2022.923905

**Published:** 2022-06-03

**Authors:** Sergio Serrano-Villar, Santiago Moreno

**Affiliations:** ^1^ Department of Infectious Diseases, Hospital Universitario Ramón y Cajal, Facultad de Medicina, Universidad de Alcalá, IRYCIS, Madrid, Spain; ^2^ CIBER de Enfermedades Infecciosas (CIBERInfec), Instituto de Salud Carlos III, Madrid, Spain; ^3^ Department of Medicine, University of California, San Francisco (USFC), San Francisco, CA, United States

**Keywords:** HIV, inflammation, C reactive protein (CRP), D-dimer (DD), antiretroviral therapy (ART)

We thank Llibre et al. for the opportunity to open a pertinent discussion (1). Given the high virologic efficacy of current ART regimens, other differentiating factors such as the effects on immune activation markers in randomized trials and cohort studies with some ART switches (2–5) are attracting attention. However, inflammation is slow to change in virally suppressed individuals (6), and our data suggests that the period typically evaluated in clinical trials risks missing potentially relevant differences.

No observational study is free of residual confounding. Would it be better to have clinical trials assessing the consequences of reducing the number of antiretrovirals after many years of follow-up? Yes. Will we see this data? Unlikely. Our study, however, offers the possibility of exploring the impact of switching to two-drug regimens (2DR) on long-term inflammation in a real-life scenario in which ART suboptimal adherence, a driver of inflammation (7) and mortality (8), is expected to be higher than in randomized trials and over a longer follow-up.

Given the data that ART adherence affects inflammation and prognosis, even in virally suppressed individuals (7, 8), the differences in inflammatory markers might be easier to detect in real-life settings than in clinical trials. Noteworthy, because we excluded NNRTI-based 3DR, most of the 3DR regimens analyzed were not available as single-table regimens, so it is unlikely that subjects in the 3DR arm received less complex regimens favoring a better adherence. The rates of low-level viremia and virological failure were higher with 3DR, suggesting that adherence was lower in participants with 3DR, as expected in an observational study. Clinicians would be less likely to reduce the number of drugs in patients with less adherence.

The authors criticize that the fraction of patients who initiated ART in the early years of the study (2005–2009) and switched to 2DR would represent a highly selected population, not comparable to that remaining on 3DR (1). However, this was not the case in our study (9): all participants switched to 2DR after 2010. Indeed, only a minor fraction of participants in the whole cohort started ART before 2010 (**Figure 1**).

**Figure 1:**
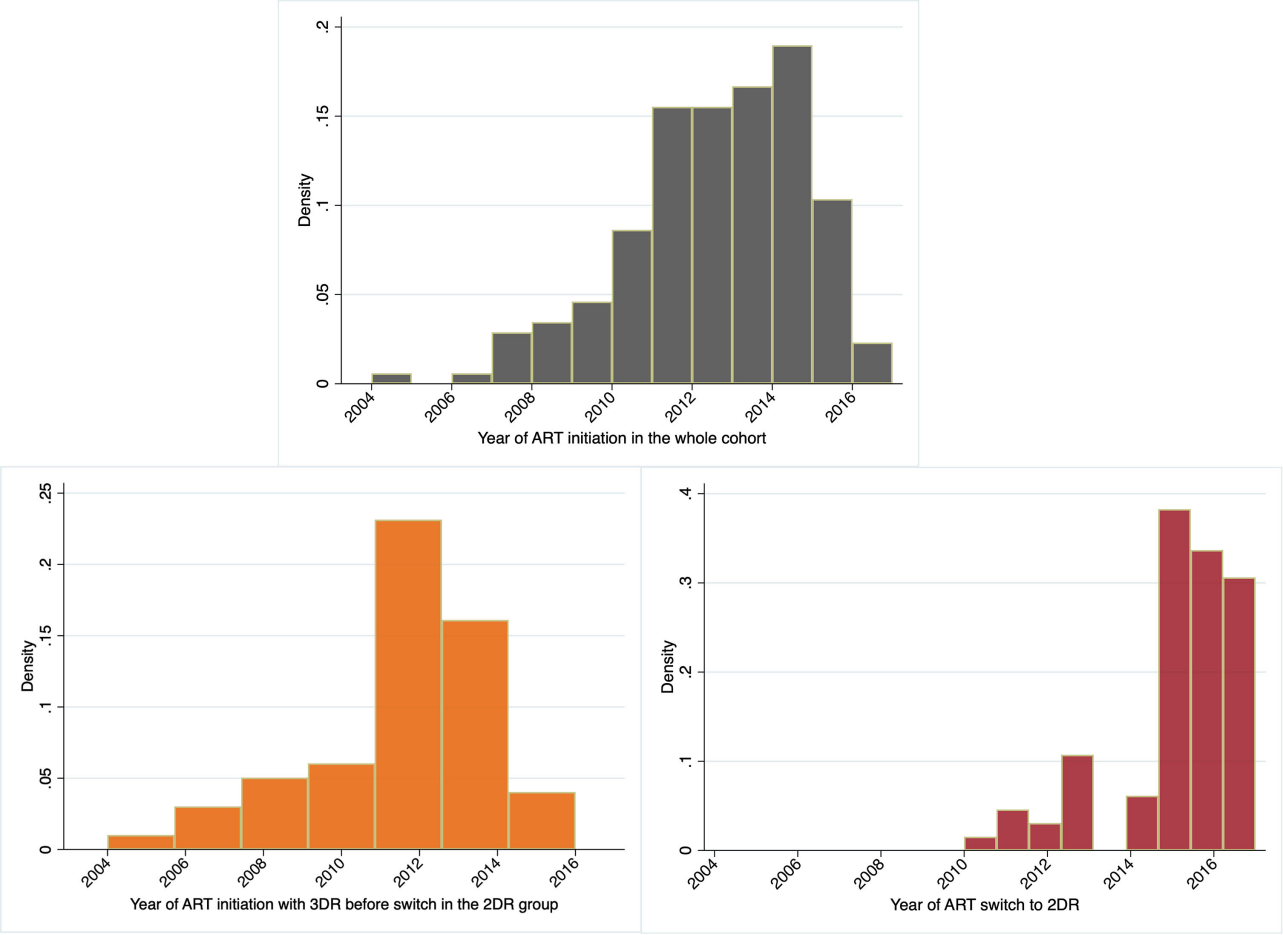
The histograms depict the percentage of participants initiating ART in the whole cohort, the 2-drug regimen group (2DR), and the year of ART switch in the 2DR group.

The authors raise a concern that our results may not be extrapolated to 2DR regimens based on dolutegravir (1). While our study is not powered to subanalyze the different 2DR combinations, 74.2% of patients in the 2DR group were receiving dolutegravir-based 2DR (either with lamivudine or rilpivirine), and the biomarker trajectories were similar in subjects with 2DR based on protease inhibitors or dolutegravir (Figure 3 of 9). Hence, most of the effect observed in the 2DR group was driven by observations from individuals who switched to dolutegravir plus lamivudine or rilpivirine, reflecting the current clinical recommendations (10, 11).

A significant contribution of our study was the possibility of assessing differences in inflammatory biomarkers during a more extended period than in previous studies, in which the power to detect differences might be limited by the short period evaluated. As we learned from the experiences using dolutegravir in monotherapy (12, 13), even under this inferior regimen, a readily measurable virological event such as a viral rebound can be slow to occur. Llibre et al. (1) noted that we have recently reported a similar rate of CD4/CD8 ratio recovery after 48 weeks of dolutegravir plus lamivudine versus dolutegravir or bictegravir-based 3DR in naïve PLWH (14). These results are reassuring, but we could only evaluate differences after 48 weeks of ART, and as we previously showed, the CD4/CD8 ratio correlates poorly with inflammatory biomarkers (15). We agree that the SWORD, TANGO, and SALSA inflammatory marker subanalyses did not yield worrisome data, as we mentioned in the discussion. However, our study could evaluate a longer period, with a median of 5.3 years in the 2DR group, allowing us to detect differences that might go unnoticed during shorter evaluations. Significantly, our study appreciates differences only after a median of 3 years after ART switch during a period of 2DR vs. 3DR that SWORD, TANGO, and SALSA could not evaluate (4, 16–18).

In our view, SWORD, TANGO, and SALSA inflammatory subanalyses have encouraged further research (4, 16–18). In these studies, the batch effects inherent to have pooled the temporal observations to be reported separately might have introduced a significant risk of observation bias, challenging the opportunity to detect consistent patterns of changes during the follow-up. In our case, the samples were carefully grouped to pool the temporal observations of each subject and achieve a similar group representation in each batch (9). We observed a consistent pattern of change for IL-6, hs-CRP, and D-dimer. Llibre et al. claim that these biomarkers strongly correlate between them in previous studies (1). To the best of our knowledge, this is not the case. Even in the studies they reference (19–22), the correlations between the inflammatory biomarkers are, at most, modest, in keeping with those reported in our manuscript (9).

We agree and advocate that we should not only rely on statistical significance to guide the interpretations of the effects of ART strategies on inflammation, especially when generated as *post hoc* analyses in large-scale studies powered to detect differences of uncertain clinical relevance. Also, we should not confer the same prognostic relevance to every inflammatory biomarker measured in these studies. For example, while the evidence linking sVCAM-1 with clinical events in the general population or during treated HIV is scarce, IL-6 is arguably the most robust inflammatory biomarker linked with all-cause mortality in PLWH (20, 22–27) and also predicts mortality risk in the general population (28). Importantly, each biomarker—IL-6, D-dimers, and CD4/CD8 ratio— seem to independently contribute to the risk prediction (29), arguing that each biomarker reflects unique pathogenic pathways. When interpreting these patterns, we should also consider the effect sizes. For example, the magnitude of sCD14 decreases observed in TANGO at week 48 was minimal (3%, treatment ratio 0.97) at week 48 (18), compared to a more considerable and also significant increase in IL-6 levels (16%, treatment ratio 1.16) that persisted at week 144 after switching to DTG/3TC vs. staying on TAF-based triple therapy [statistical significance reported in a conference (30), but not in the published manuscript (4)]. We agree that we must be extremely cautious with these observations. Accordingly, we must demand a transparent reporting of the methods and statistical analyses performed and a fair interpretation of the results. Assuming that a smaller sCD14 decrease in the 2DR arm in TANGO attenuates the concerns raised by the larger IL-6 increases is, at best, a simplistic interpretation.

Whether the differences reported in inflammation between ART choices are clinically relevant or not remains an open question. We appreciate the effort by the RESPOND European-Australian consortium to assess the rates of clinical outcomes with 3DR compared to 2DR (31). However, there was high heterogeneity in the ART combinations in this cohort, including regimens not currently recommended in clinical guidelines (10, 11) and no inflammatory markers were measured. Thus, no associations between the risk of outcomes and the differential effects of inflammation could be established. Furthermore, the study was likely underpowered to detect the differences predicted by the model. The incidence ratio of clinical events on 2DR compared to 3DR was 1.28 (95%CI 0.88-1.87), indicating that there was a 28% higher incidence risk of adverse outcomes that was not statistically significant. The wideness of the confidence interval does not allow concluding that the risk is similar. We have recently shown in a Markov model study that to detect differences on clinical outcomes between 2DR and 3DR based on the effects of IL-6 and D-dimers on severe non-AIDS events previously reported (27) and the IL-6 and D-dimer changes appreciated in TANGO (30) and our study (9), a larger sample size or a longer follow-up will be needed (32).

Our work was intended and presented as hypothesis generating rather than hypothesis testing, and we hope that this commentary will help to avoid misinterpretations of our findings. We believe that whether the number and type of antiretrovirals or the method of delivery differentially affect inflammation is far from being settled, especially in the scenario of long-term treatment. It is still unclear what are the mechanisms driving differences in long-term immune activation between ART choices. Distinct effects on weight change, tolerability impacting ART adherence, or particular drug distribution to lymphoid tissues resulting in low-level production of viral proteins eliciting immune activation could play a role. While translational studies will help understand the mechanisms, large cohort studies and randomized clinical trials designed to address these knowledge gaps (33, 34) are ongoing and will enable move the field forward.

## Author Contributions

SS-V and SM conceptualized the work, SS-V wrote the first draft, SM reviewed and approved the final manuscript.

## Conflict of Interest

Outside the submitted work, S.S-V. reports personal fees from ViiV Healthcare, Janssen Cilag, Gilead Sciences, and MSD as well as non-financial support from ViiV Healthcare and Gilead Sciences and research grants from MSD and Gilead Sciences. SM reports grants, personal fees and non-financial support from ViiV Healthcare, personal fees and non-financial support from Janssen, grants, personal fees and non-financial support from MSD, grants, personal fees and non-financial support from Gilead, outside the submitted work.

## Publisher’s Note

All claims expressed in this article are solely those of the authors and do not necessarily represent those of their affiliated organizations, or those of the publisher, the editors and the reviewers. Any product that may be evaluated in this article, or claim that may be made by its manufacturer, is not guaranteed or endorsed by the publisher.
